# Supplementary material for: Multivalent interactions essential for lentiviral integrase function
Source: Nat Commun. 2022 May 3;13:2416. doi: 10.1038/s41467-022-29928-8 (PMC9065133; doi:10.1038/s41467-022-29928-8)
Supplement: Supplementary file 1 — Supplementary Information [file 41467_2022_29928_MOESM1_ESM.pdf]

Supplementary Information

Multivalent interactions essential for lentiviral integrase function

Allison Ballandras-Colas, Vidya Chivukula, Dominika T. Gruszka, Zelin Shan, Parmit K. Singh, Valerie E. Pye, Rebecca K. McLean, Gregory J. Bedwell, Wen Li, Andrea Nans, Nicola J. Cook, Hind J. Fadel, Eric M. Poeschla, David J. Griffiths, Javier Vargas, Ian A. Taylor, Dmitry Lyumkis, Hasan Yardimci, Alan N. Engelman, and Peter Cherepanov

Supplementary Figures: 12
Supplementary Tables: 5

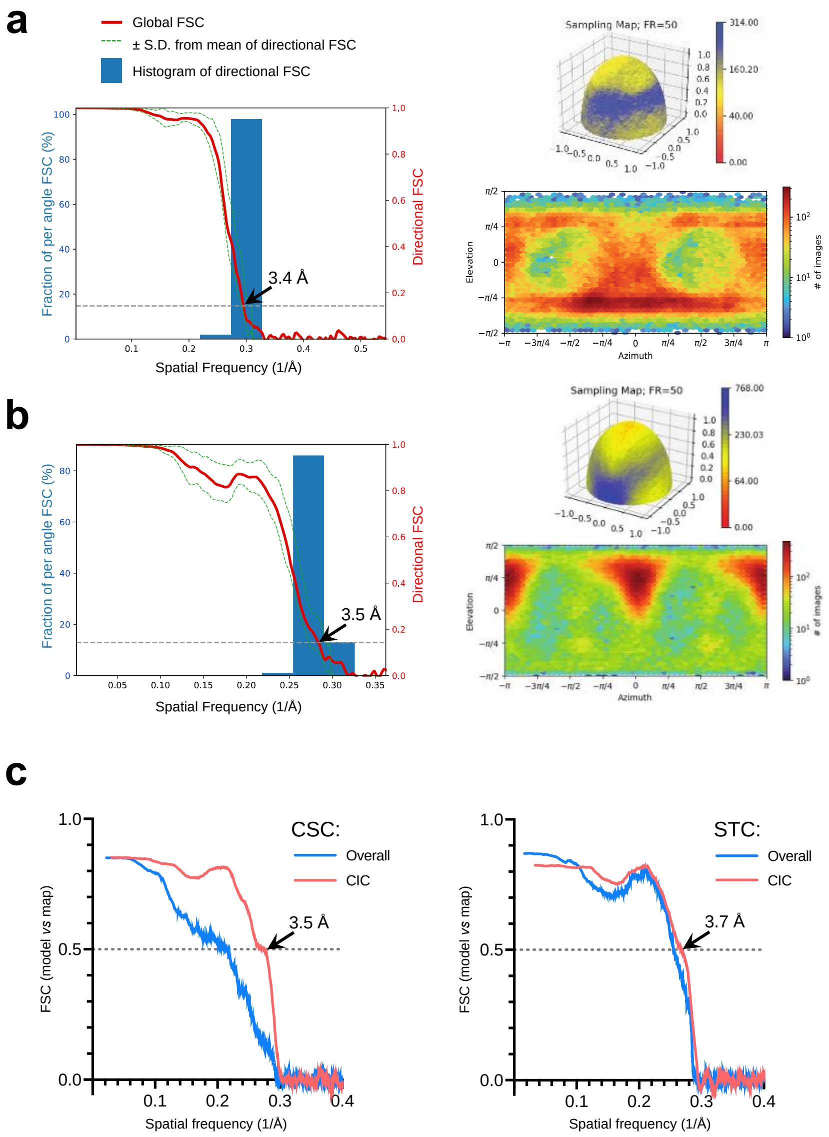

Supplementary Figure 1. Directional resolution of cryo-EM reconstructions, particle views bias, and model fit indicators. (a) Directional resolution metrics and estimated projection distributions for the MVV CSC. Left: the output generated by the 3DFSC software [82] showing the global FSC curve (thick red line), boundaries of the directional FSCs (± 1 standard deviation, dotted green lines), and a histogram of directional FSC values (blue bars). Estimated nominal resolution using the fixed FSC 0.143 threshold is indicated with a black arrowhead. Top right and bottom right show the surface sampling plot [89] and estimated Euler angle distribution for the set of particles contributing to the 3D reconstruction. **(b)** Same as in panel A for MVV STC. **(c)** Model vs map FSC plots for MVV CSC (left) and STC (right). Blue and orange lines represent FSCs for the entire model and the CIC, respectively.

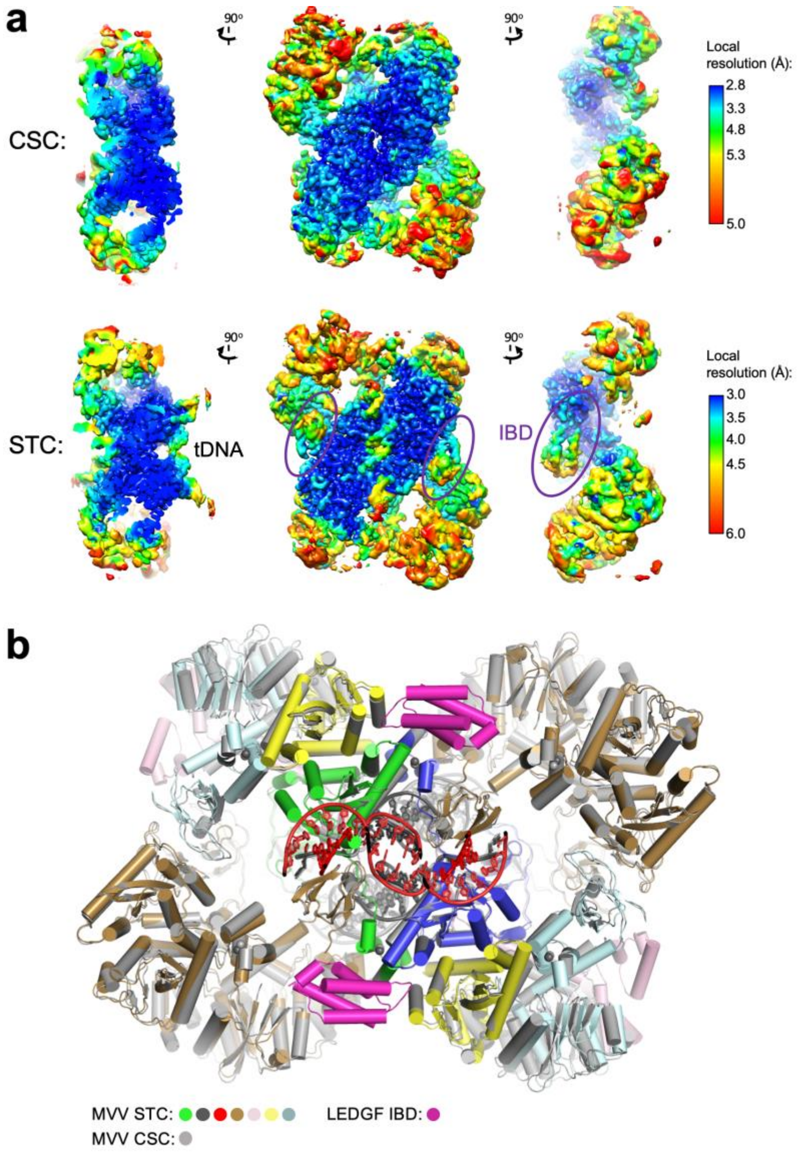

Supplementary Figure 2. Local resolutions of cryo-EM reconstructions and comparison of the final CSC and STC models. (a) Local resolution distributions for CSC (top) and STC (bottom) cryo-EM reconstructions indicated with color code corresponding to the key on the right. The maps are shown in three orientations; volumes on the left are shown as orthoslices. The map features corresponding to LEDGF/p75 IBD are indicated with purple ovals. **(b)** Superposition of the refined CSC and STC models. The protein subunits are shown as cartoons, with helices as tubes and DNA as sticks. STC is colored as in Fig. 1a and CSC in grey.

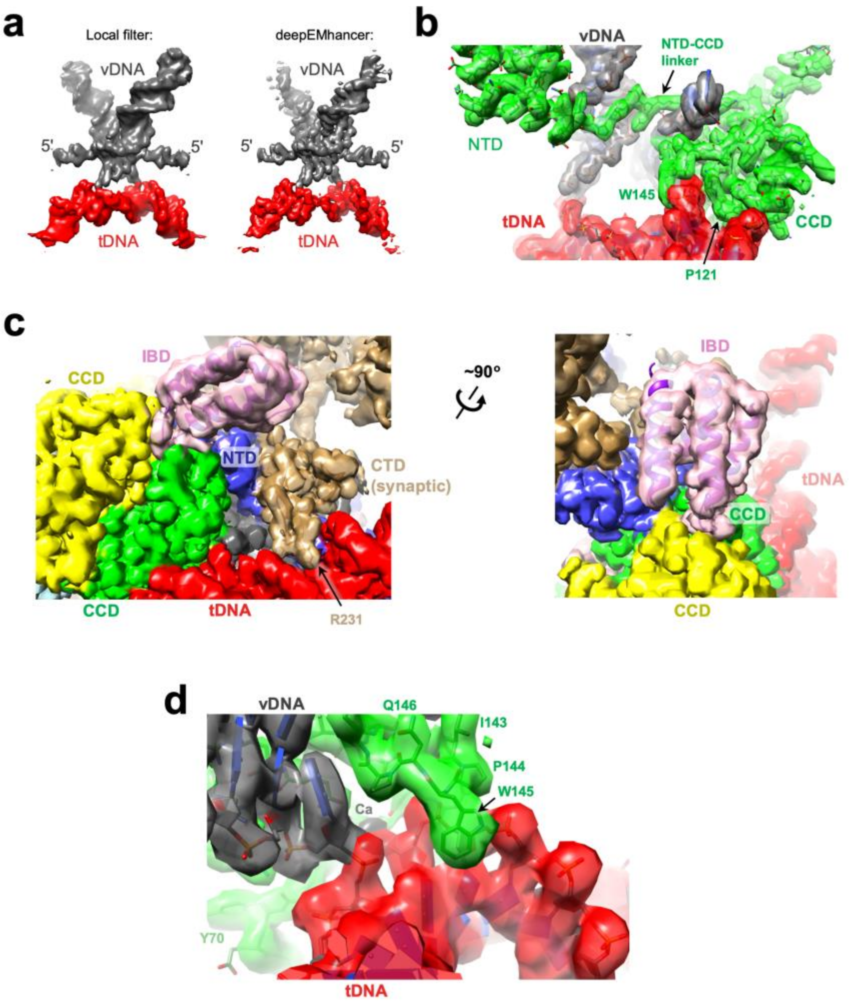

Supplementary Figure 3. Examples of the STC cryo-EM map (a) The region corresponding to the vDNA-tDNA synapse from the STC cryo-EM map sharpened using local filtering in cryoSPARC (left) or using DeepEMhancer [93] (right). 5'-unpaired vDNA nucleotides are indicated to the left. **(b-d)** Selected regions of the STC volume sharpened using DeepEMhancer. Selected structural elements, including the NTD-CCD linker (panel b), and amino acid residues are indicated.

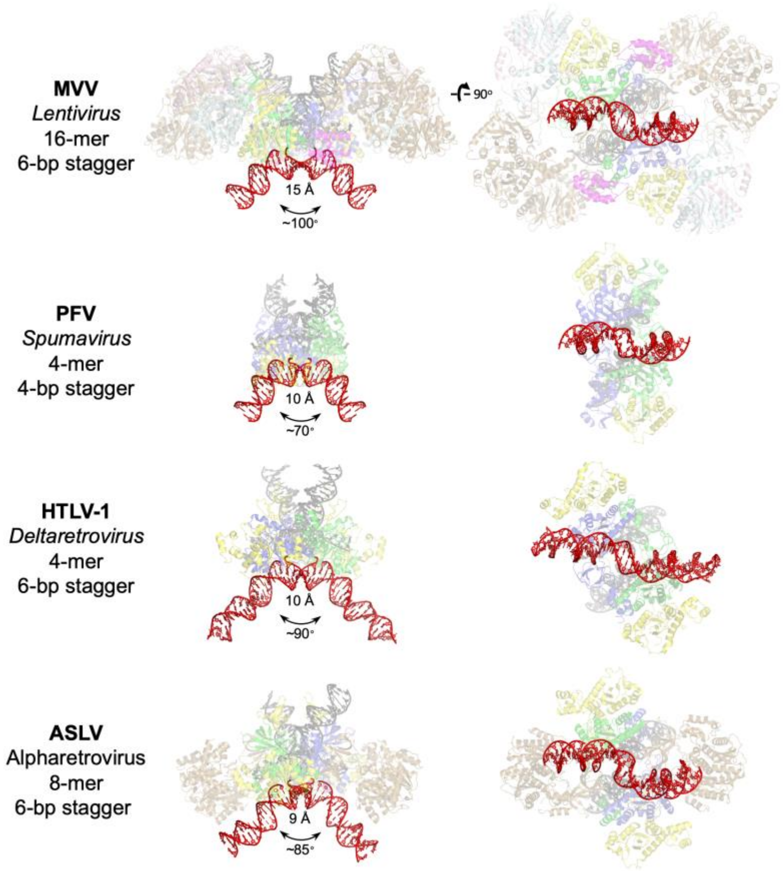

Supplementary Figure 4. Conformations assumed by tDNA within diverse retroviral STCs. Protein subunits and DNA chains in each structure are shown in cartoons and sticks, respectively. The tDNA portions are shown opaque (red), with the rest of the structures semi-transparent. The STC structures shown are from MVV (this work), PFV (PDB ID 4BAC)[10], HTLV-1 (PDB ID 6VOY)[15], and ASLV (PDB ID 5EJK)[12]. Each structure is shown in two orthogonal orientations. The virus species, genus, corresponding IN multimeric state, and integration stagger (bp) are reported to the left. The minor groove width at the site of integration and the angle between tDNA arms are reported under each left panel.

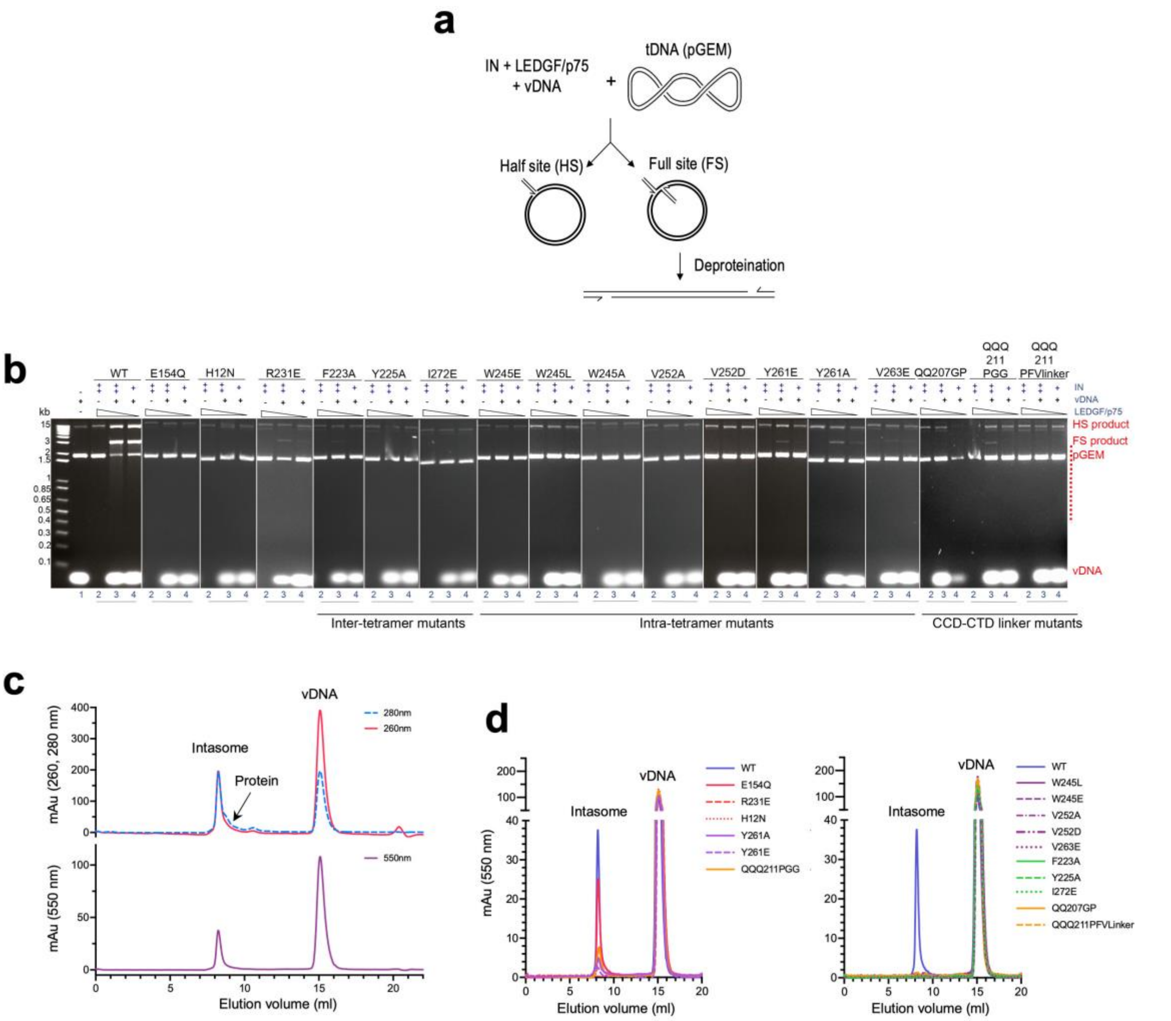

Supplementary Figure 5. ***In vitro* activities of MVV IN variants. (a)** Schematic of the strand transfer assay, which utilizes double-stranded oligonucleotides matching the processed 3' end (U5) of the MVV reverse transcript and supercoiled plasmid (pGEM) as mimics of vDNA ends and target DNA, respectively. The assay allows detection of two types of strand transfer products: full-site, resulting from insertion of pairs of vDNA ends into opposing strands of tDNA and half-site, resulting from insertion of a single vDNA end. When resolved by agarose gel electrophoresis, the full- and half-site products co-migrate with linearized and open-circular forms of the plasmid, respectively. Note that multiple full-site strand transfer events result in fragmentation of target DNA, giving rise to smearing that becomes pronounced at higher MVV IN and LEDGF/p75 inputs. **(b)** Strand transfer activities of MVV IN variants. WT and mutant INs at a concentration of 1.1 µM (lanes 2 and 3) or 0.55 µM (lanes 4) were incubated with 1.5 µM (lanes 2 and 3) or 0.75 µM (lanes 4) LEDGF/p75, with (lanes 3 and 4) or without (lanes 2) 20 µM vDNA in the presence of 7.5 ng/µL pGEM target DNA. Lane 1 contained a mock reaction without IN and LEDGF/p75. Deproteinized reaction products were separated in 1.5% agarose gels and detected by staining with ethidium bromide. Migration positions of the reaction products, vDNA and pGEM are indicated on the right of the gel; positions of DNA molecular size markers (kb) are shown on the left. The results are representative of experiments repeated three times. **(c)** Analysis of CSC intasome assembly containing WT MVV IN and Cy3-labeled vDNA oligonucleotide by size exclusion chromatography. UV (wavelengths 260

and 280 nm) and visible light (550 nm) absorbance of the eluate is plotted on the top and bottom, respectively. Elution positions of the intasome, protein (IN-LEDGF complexes) and vDNA are indicated. **(d)** Elution profiles of intasome assembly reactions containing WT and two sets of mutant MVV INs and Cy3-labeled vDNA. Chromatograms on the left represent full elution profiles of the assembly reactions shown in Fig. 3d. Source data are provided as a Source Data file.

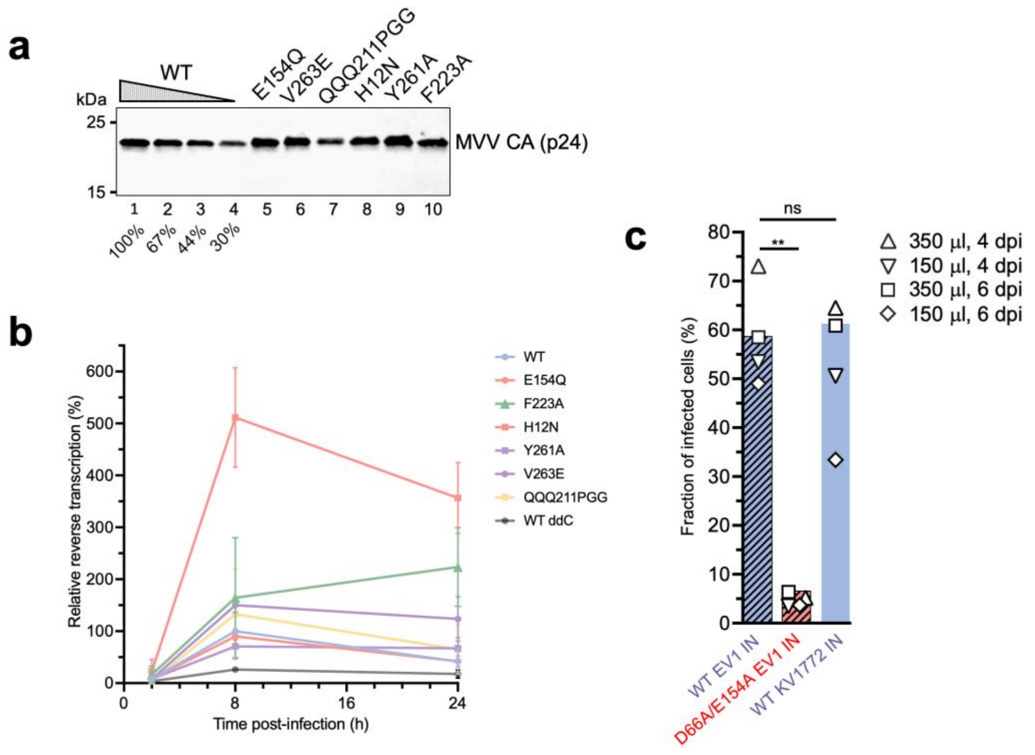

Supplementary Figure 6. Capsid protein and reverse transcription profiles of MVV vectors. (a) MVV vector preparations containing 0.5 mU associated RT activity were separated in lanes 1 (WT) and 5-10 (for indicated mutants). Lanes 2, 3, and 4 contained serial 1.5-fold dilutions of the WT sample. Mature MVV capsid/p24 protein was detected by Western blotting with a rabbit polyclonal antibody and IRDye 800CW-conjugated secondary antibody. The results are representative of experiments repeated three times. **(b)** Levels of late reverse transcription products measured in HEK293T cells at 2, 8 and 24 h post-infection with WT and IN mutant MVV vectors. Line plots represent means, and standard deviations were derived from n=3 biological replicates. The grey line reports vDNA levels in cells infected with WT MVV in the presence of 100 µM ddC. **(c)** Infectivity of GFP-reported MVV vectors produced using Gag-Pol constructs with the IN-coding region derived from EV1 or KV1772 MVV isolate. HEK293T cells were infected with equal 350 µL (corresponding to 16.8 mU of associated RT activity) or 150 µL of WT or active site mutant (D66A/E154A) EV1, or WT KV1772 IN vector. GFP-positive cells were counted 4 or 6 d post-infection by flow cytometry. Results of four individual measurements are indicated with symbols, and bars represent fractions of GFP-expressing cells 4 days post-infection with 150 µL virus. Statistical significance was tested using paired two-tailed Student's t-test (ns, non-significant, $p = 0.2$; **, $p = 0.002$). Source data are provided as a Source Data file

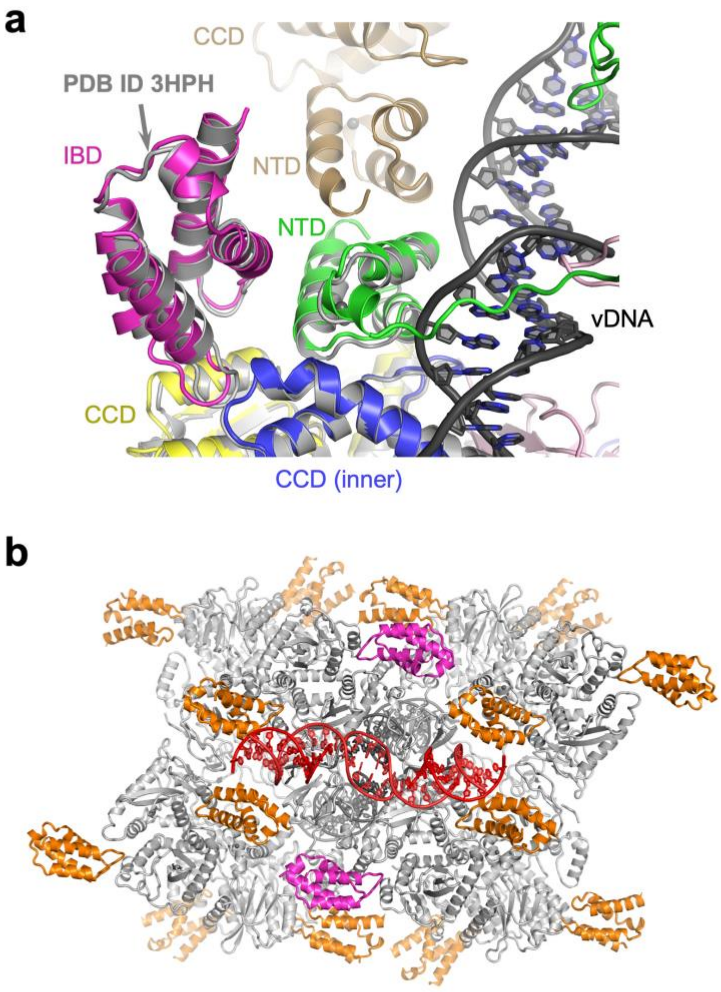

Supplementary Figure 7. MVV intasome - LEDGF/p75 IBD interactions. (a) Superposition of the MVV STC (shown as cartoons and colored as in Fig. 1A) and MVV IN-LEDGF/p75 co-crystal structure (grey cartoons; PDB ID 3HPH)[33]. **(b)** A model of the MVV intasome (grey) with every potential LEDGF/p75 IBD binding site occupied. The two IBDs resolved in the STC structure are shown in magenta, while fourteen modeled IBDs are in orange.

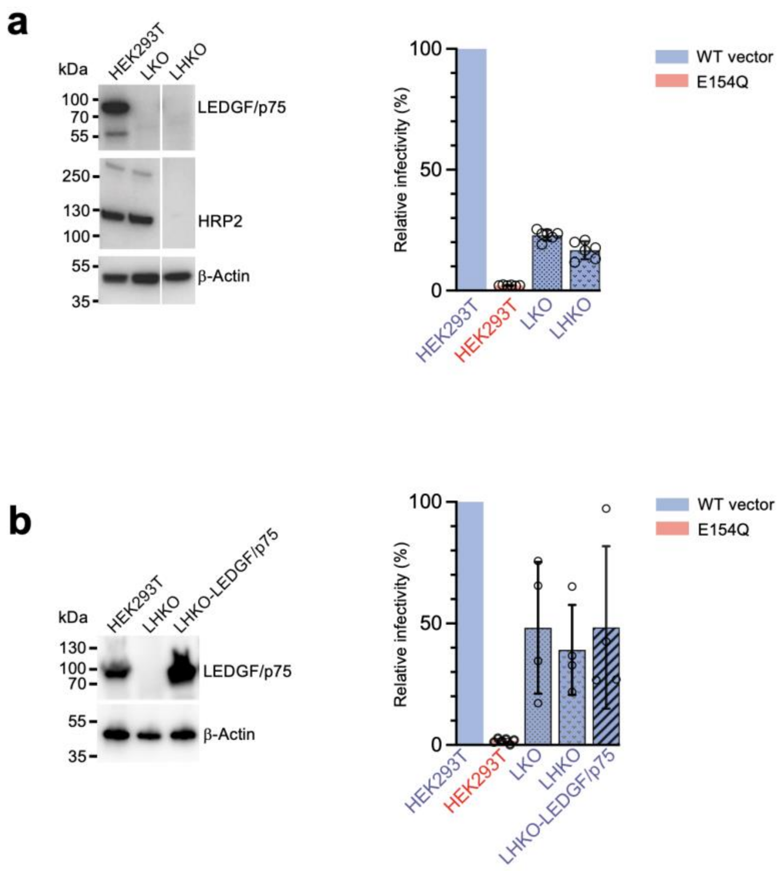

Supplementary Figure 8. MVV vector infection of gene-modified human cells. (a) Infectivity of MVV vector in human LKO and LHKO cells. Left: Western blotting analysis of total cell extracts of LKO, LHKO, and parental HEK293T cells with anti-LEDGF/p75 (top), anti-HRP2 (middle) and anti-β-actin antibodies (used as a loading control, bottom). Right: HEK293T, LKO, and LHKO cells were infected with WT or E154Q MVV vectors, normalized by associated RT activity and encoding a luciferase reporter. Luciferase expression was measured 7 d post-infection; bar plots represent mean values relative to WT, which was set to 100% for each replicate series. Standard deviations were calculated from n=6 biological replicates; open circles are the individual measurements obtained in replicate experiments. **(b)** Overexpression of LEDGF/p75 in LHKO cells does not rescue MVV vector infectivity. Left: Western blot analysis of whole cell extracts of LHKO, LHKO-cells prior and after overexpression of ovine LEDGF/p75 and parental HEK293T cells with anti-LEDGF/p75 (top) and anti-β-actin antibodies (used as a loading control, bottom). Right: The cells were infected with WT or E154Q IN MVV vectors normalized by RT activity and encoding a luciferase reporter. Expression of luciferase was measured 7 d post-infection. Bar plots represent mean values relative to WT, which was set to 100% within each replicate series. Standard deviations were calculated from n=4 biological replicates; open circles are individual measurements obtained in replicate experiments. Source data are provided as a Source Data file.

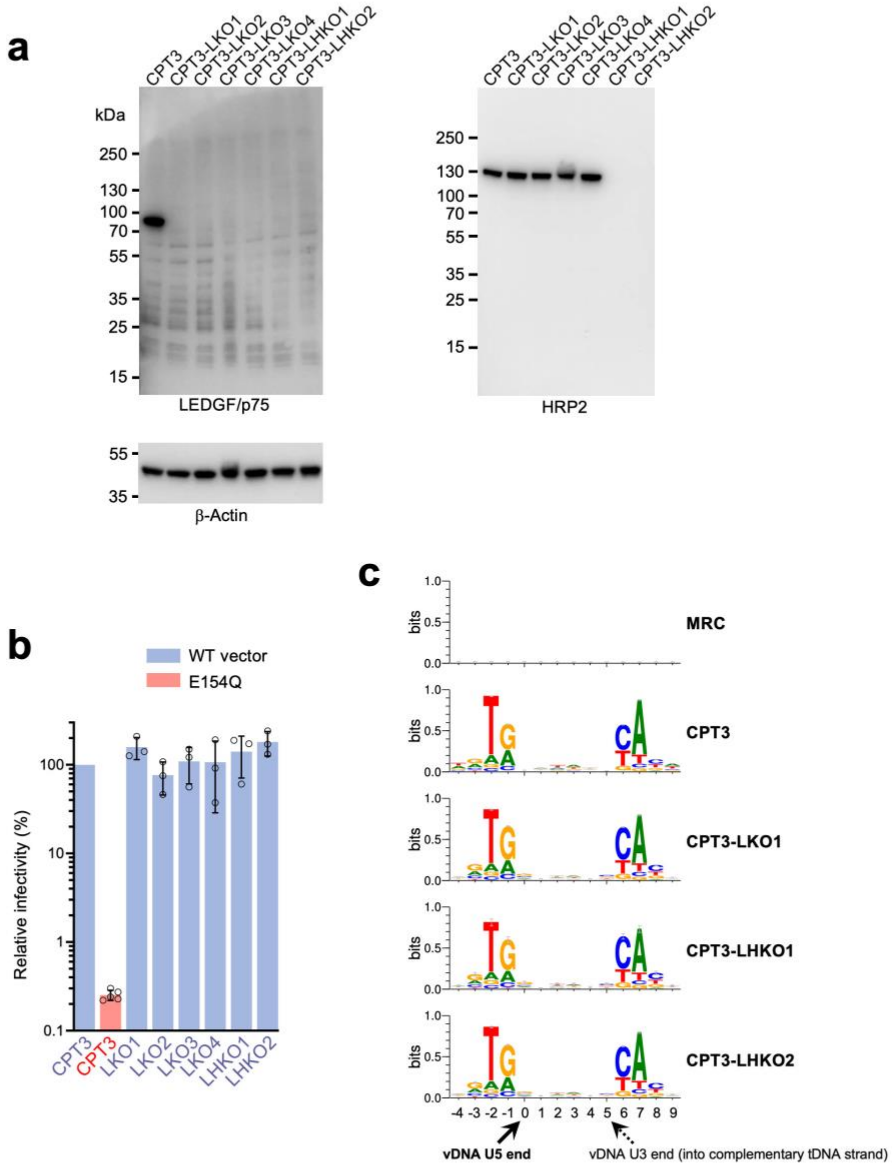

Supplementary Figure 9. MVV infection of WT and gene-modified ovine cells. (a) Ablation of LEDGF/p75 and HRP2 in CPT3 cells transfected with CRISPR-Cas9 RNPs targeting ovine *PSIP1* and *HDGFL2* genes (Supplementary Table 5). Equal amounts of whole cell extracts (20 µg total protein) from cells transfected with combinations of RNPs were analyzed by Western blotting with anti-LEDGF/p75 and anti-HRP2 antibodies. **(b)** MVV vector infectivity in ovine cells in the absence of LEDGF/p75 and HRP2. Ovine parental CPT3 cells or CPT3-LKO and CPT3-LHKO cells (as indicated) were infected with luciferase-reporter WT or E154Q IN MVV vectors normalized by RT activity. Luciferase expression was measured 7 d post-infection. Open circles indicate individual replicate measurements. Bars represent means; standard deviations were calculated from n=3 biological replicates, except for E154Q, infectivity of which was measured 5 times. **(c)** Sequence logos showing local target nucleotide sequence preferences of MVV vector determined from mapped integration sites in ovine CPT3, CPT3-LKO1, CPT3-LHKO1, and CPT3-LHKO2. The mock logo (top) was generated using alignment of matched random control (MRC) sites in ovine genome. Source data are provided as a Source Data file.

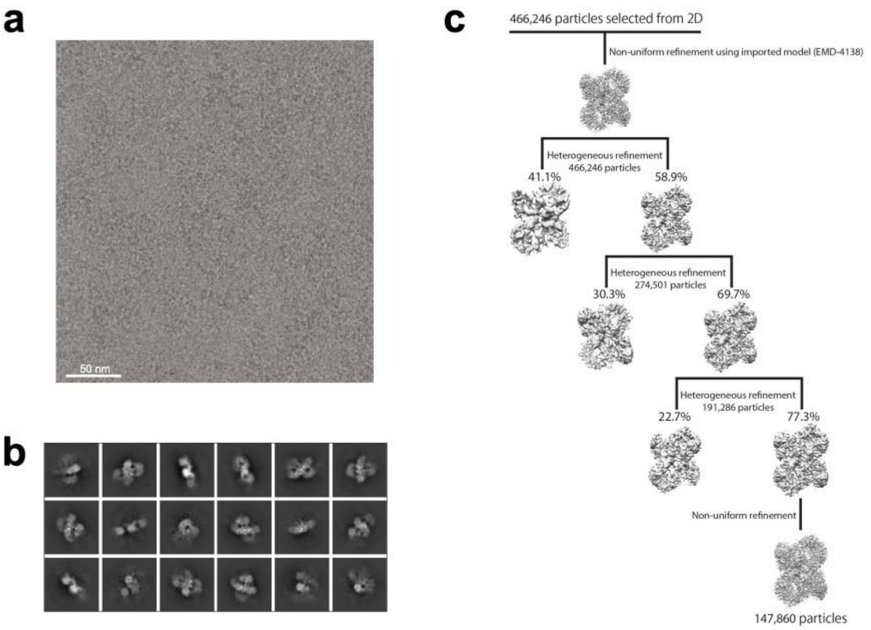

Supplementary Figure 10. MVV CSC intasome images and classification. (a) Example of an image of MVV CSC particles vitrified in amorphous ice in open holes. The image is representative of 2,295 micrograph movies acquired (Supplementary Table 1; see Methods for details). **(b)** 2D class averages of MVV CSC particles. **(c)** 3D classification workflow utilizing iterative cycles of heterogeneous refinement and non-uniform refinement to improve the quality of the map; 147,860 particles remained for the final non-uniform refinement and reconstruction.

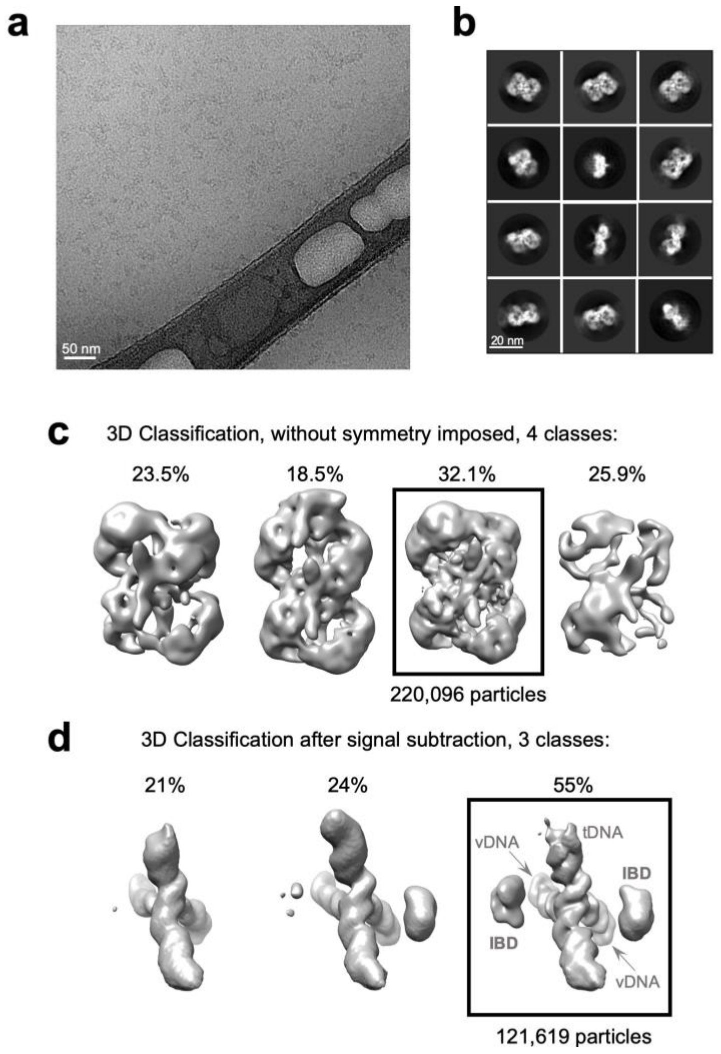

Supplementary Figure 11. MVV STC intasome images and classification. (a) Example of an image of MVV STC particles vitrified in amorphous ice and supported by ultrathin carbon. The image is representative of 11,760 micrograph movies acquired (Supplementary Table 1; see Methods for details). **(b-c)** 2D and 3D class averages of MVV STC particles. The 3D class containing 220,096 particles (boxed in panel c) was taken for 3D classification after subtracting IN-derived signal. **(d)** Results of 3D classification following IN signal subtraction from STC particle images. The 3D class containing 121,619 particles (boxed) with good IBD occupancy at two positions was used for the final refinement and reconstruction.

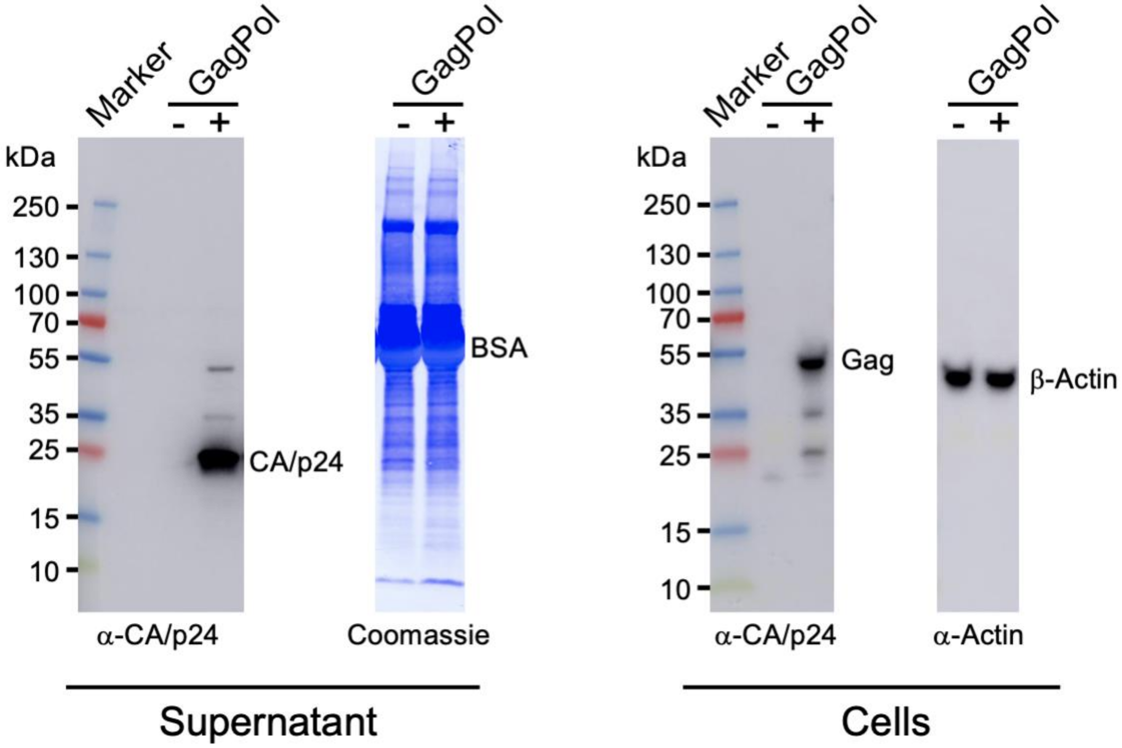

Supplementary Figure 12. Validation of the rabbit polyclonal anti-MVV CA/p24 antibody. HEK293T cells were transfected with pCVW-CG-Luc, pCMV-VMV-Rev, and pMD2.G with (+ GagPol) or without (- GagPol) viral packaging construct pCAG-MV-GagPol-IN^{KV1772}-CTEx2, using a total of 35 μg plasmid DNA per 15-cm dish. Cell culture supernatant and cells were harvested 36 h post-transfection. Supernatants (6 μl, concentrated 160-fold by ultracentrifugation at 100,000 g for 90 min), whole cell extracts (50 μg total protein) and pre-stained protein ladder (Marker) were separated by SDS polyacrylamide gel electrophoresis and transferred to polyvinylidene fluoride membranes. The membranes were probed with polyclonal rabbit anti-CA/p24 (batch AB2670) or HRP-conjugated rabbit monoclonal anti-β-actin antibody (clone 13E5, Cell Signaling Technology, product code 5125) or stained with Coomassie Blue. Migration positions of processed CA/p24, bovine serum albumin (BSA), unprocessed MVV Gag polyprotein and β-actin are indicated to the right of each blot image.

Supplementary Table 1. Cryo-EM data collection, image processing and model refinement.

	CSC	STC + LEDGF/p75
**Data collection**		
Microscope, operating voltage	Talos Arctica, 200 keV	Titan Krios, 300 keV
Detector	Gatan K2	Gatan K2
Magnification (nominal)	45,000	36,232
Calibrated pixel size (Å)	0.92	1.38
Underfocus range (nominal, µm)	1.0-3.0	1.5-4.5
Stage tilt (°)	40	0
Number of frames per movie	100	50
Total electron fluence (e/Å^2)	43.6	50.4
Automation software	Leginon	EPU
Total number of movies used	2,295	11,760
**Reconstruction**		
Software for 2D classification	cryoSPARC-2	Relion-2.1
Software for 3D classification	cryoSPARC-2	Relion-2.1
Software for reconstruction	cryoSPARC-2	cryoSPARC-2
Number of initially extracted particles	926,176	2,022,321
Number of refined particles	147,860	121,619
Symmetry imposed	*C2*	*C2*
Resolution global (FSC 0.143, Å)	3.4	3.5
SCF value [a]	0.76	0.84
**Model refinement**		
Software for real-space refinement	Phenix dev-4213-000	Phenix dev-4213-000
Model composition		
Non-hydrogen atoms	32,156	35,617
Protein residues	3,802	4,031
Nucleic acid bases	64	140
Metal ions (Zn/Ca)	12/2	12/0
B factors (Å^2)		
Protein	262.6	198.1
Nucleic acid bases	146.9	199.4
Metal ions (Zn/Ca)	148.6	270.72
Real-space correlation coefficient	0.70	0.77
R.m.s. deviations		
Bond lengths (Å)	0.002	0.002
Bond angles (°)	0.51	0.51
Validation[c]		
MolProbity score	1.51	1.48
Clash score	7.51	7.25
Poor rotamers (%)	0	2
Ramachandran plot quality (%) [b]		
Favoured	97.49	97.63
Disallowed	0	0

[a] Based on the nominal Euler angle distribution

[b]Assessed using MolProbity [100].

Supplementary Table 2. Descriptive statistics for LEDGF/p75-Surf649 photobleaching experiments [a].

	0.2 M NaCl	0.5 M NaCl	1 M NaCl
N	605	745	516
Minimum	1	1	1
25% Percentile	5	2	1
**Median**	**6**	**4**	**2**
75% Percentile	8	5	3
Maximum	16	12	7
Mean ± Std. Error	6.41 ± 0.10	3.78 ± 0.07	2.39 ± 0.06
Std. Dev. ± Std. Error	2.45 ± 0.10	1.79 ± 0.07	1.36 ± 0.06
**Intasome: LEDGF binding stoichiometry**	**1 : 6**	**1 : 4**	**1 : 2**

[a] Source data are provided as a Source Data file.

Supplementary Table 3. Statistical significance tests of HIV-1 and MVV integration site distributions in human cells.

	MRC	HIV-1 in HEK293T	HIV-1 in LKO	MVV in HEK293T	MVV in LKO
Integration in TUs [a]					
HIV-1 in HEK293T	$<10^{-300}$				
HIV-1 in LKO	$<10^{-300}$	$<10^{-300}$			
MVV in HEK293T	$<10^{-300}$	$<10^{-300}$	$2\cdot10^{-40}$		
MVV in LKO	0.01	$<10^{-300}$	10^{-45}	10^{-77}	
MVV in LHKO	$5\cdot10^{-35}$	$<10^{-300}$	10^{-227}	$<10^{-300}$	0.2
Integration close TSSs [a]					
HIV-1 in HEK293T	$3\cdot10^{-5}$				
HIV-1 in LKO	$<10^{-300}$	10^{-210}			
MVV in HEK293T	10^{-81}	$2\cdot10^{-44}$	$<10^{-300}$		
MVV in LKO	10^{-15}	$2\cdot10^{-11}$	$2\cdot10^{-5}$	$3\cdot10^{-26}$	
MVV in LHKO	10^{-159}	10^{-80}	$5\cdot10^{-22}$	$2\cdot10^{-278}$	0.7
Integration near CpG islands [a]					
HIV-1 in HEK293T	$6\cdot10^{-59}$				
HIV-1 in LKO	$<10^{-300}$	$2\cdot10^{-184}$			
MVV in HEK293T	10^{-157}	10^{-207}	$<10^{-300}$		
MVV in LKO	10^{-16}	10^{-4}	$2\cdot10^{-9}$	$3\cdot10^{-33}$	
MVV in LHKO	10^{-223}	$3\cdot10^{-52}$	10^{-29}	$<10^{-300}$	0.1
Integration near cLADs [a]					
HIV-1 in HEK293T	$<10^{-300}$				
HIV-1 in LKO	$<10^{-300}$	10^{-129}			
MVV in HEK293T	$<10^{-300}$	$<10^{-300}$	$<10^{-300}$		
MVV in LKO	0.2	$3\cdot10^{-154}$	$3\cdot10^{-59}$	$2\cdot10^{-06}$	
MVV in LHKO	0.05	$<10^{-300}$	$<10^{-300}$	$2\cdot10^{-69}$	0.5
Integration near SPADs [a]					
HIV-1 in HEK293T	$<10^{-300}$				
HIV-1 in LKO	$<10^{-300}$	$<10^{-300}$			
MVV in HEK293T	$4\cdot10^{-4}$	$<10^{-300}$	$<10^{-300}$		
MVV in LKO	10^{-20}	$2\cdot10^{-135}$	10^{-23}	$4\cdot10^{-19}$	
MVV in LHKO	10^{-160}	$<10^{-300}$	$2\cdot10^{-169}$	$1\cdot10^{-149}$	0.5
Gene density near integration sites [b]					
HIV-1 in HEK293T	$<10^{-300}$				
HIV-1 in LKO	$<10^{-300}$	$<10^{-300}$			
MVV in HEK293T	$<10^{-300}$	$<10^{-300}$	$<10^{-300}$		
MVV in LKO	10^{-12}	$2\cdot10^{-289}$	10^{-73}	0.3	
MVV in LHKO	$2\cdot10^{-47}$	$<10^{-300}$	$<10^{-300}$	$4\cdot10^{-8}$	0.02
GC content near integration sites [b]					
HIV-1 in HEK293T	$6\cdot10^{-121}$				
HIV-1 in LKO	$6\cdot10^{-153}$	$8\cdot10^{-289}$			
MVV in HEK293T	$<10^{-300}$	$4\cdot10^{-84}$	$<10^{-300}$		
MVV in LKO	$4\cdot10^{-64}$	10^{-98}	$3\cdot10^{-18}$	10^{-149}	
MVV in LHKO	$3\cdot10^{-154}$	10^{-282}	0.07	$<10^{-300}$	$2\cdot10^{-14}$

[a] *P* values determined using two-sided Fisher's exact test.
[b] *P* values determined using two-sided Wilcoxon rank-sum test.

Supplementary Table 4. Statistical significance tests of MVV integration site distributions in ovine cells.

	MRC	CPT3	CPT3-LKO1	CPT3-LHKO1
Integration into TUs [a]				
CPT3	$<10^{-300}$			
CPT3-LKO1	$<10^{-300}$	$<10^{-300}$		
CPT3-LHKO1	10^{-7}	$3{\cdot}10^{-221}$	$3{\cdot}10^{-7}$	
CPT3-LHKO2	$<10^{-300}$	$<10^{-300}$	0.2	$2{\cdot}10^{-6}$
Integration near TSS [a]				
CPT3	10^{-24}			
CPT3-LKO1	$<10^{-300}$	$<10^{-300}$		
CPT3-LHKO1	$2{\cdot}10^{-55}$	$2{\cdot}10^{-43}$	0.004	
CPT3-LHKO2	$<10^{-300}$	$<10^{-300}$	0.7	0.003
Integration near CpG islands: [a]				
CPT3	$2{\cdot}10^{-181}$			
CPT3-LKO1	$<10^{-300}$	$<10^{-300}$		
CPT3-LHKO1	10^{-84}	$2{\cdot}10^{-139}$	0.008	
CPT3-LHKO2	$<10^{-300}$	$<10^{-300}$	0.002	0.07
Gene density near integration sites [b]				
CPT3	$<10^{-300}$			
CPT3-LKO1	$<10^{-300}$	$<10^{-300}$		
CPT3-LHKO1	$<10^{-300}$	10^{-4}	$2{\cdot}10^{-4}$	
CPT3-LHKO2	$<10^{-300}$	$2{\cdot}10^{-78}$	$5{\cdot}10^{-32}$	0.6
GC content near integration sites [b]				
CPT3	$<10^{-300}$			
CPT3-LKO1	$<10^{-300}$	$<10^{-300}$		
CPT3-LHKO2	$6{\cdot}10^{-274}$	$<10^{-300}$	$<10^{-300}$	

[a] *P* values determined using two-sided Fisher's exact test.
[b] *P* values determined using two-sided Wilcoxon rank-sum test.

Supplementary Table 5. Oligoribonucleotides used as crRNAs to disrupt ovine *PSIP1* and *HDGFL2*.

	Target gene	Target genomic sequence [a]	Target location [b]
crRNA 1	*PSIP1*	ttaatttatagAAACATCAA	chr2:83429226-83429245
crRNA 2	*PSIP1*	CAATGGATTCTCGACTTCAA	chr2:83429209-83429228
crRNA 3	*PSIP1*	CAACAGATGTATTGAGGCTT	chr2:83428933-83428952
crRNA 4	*PSIP1*	TATTGAGGCTTTGGATGAAC	chr2:83428924-83428943
crRNA 5	*HDGFL2*	cctacctgcagAGCCCTCCG	chr5:17145214-17145233
crRNA 6	*HDGFL2*	acctgcagAGCCCTCCGTGG	chr5:17145211-17145230
crRNA 7	*HDGFL2*	tcacCGGATTGTCAACCTTC	chr5:17145148-17145167
crRNA 8	*HDGFL2*	TGAGATCAAGTTCGCCCTGA	chr5:17145166-17145185

[a] Intron-derived sequences are given in lower case.
[b] Coordinates within oviAri4 sheep genome assembly (https://genome.ucsc.edu/).
