# Supplementary material for: Multivalent interactions essential for lentiviral integrase function
Source: Nat Commun. 2022 May 3;13:2416. doi: 10.1038/s41467-022-29928-8 (PMC9065133; doi:10.1038/s41467-022-29928-8)
Supplement: Supplementary file 3 — Description of Additional Supplementary Files [file 41467_2022_29928_MOESM3_ESM.pdf]

Description of Additional Supplementary Files

File Name: Supplementary Movie 1
Description: **Photobleaching of the MVV intasome-LEDGF-Surf649 complexes.** The recording was done in the presence of 1.0 M NaCl in the field of view presented in Fig. 4b (14.4 by 14.4 µm). The initial 5 frames show vDNA-Cy3 fluorescence (yellow), followed by the photobleaching of LEDGFSurf649 (red) over 196 frames. The movie is presented in real time (40 s total; 5 frames per second); the scale bar is 3.0 µm
