# Supplementary material for: Multivalent interactions essential for lentiviral integrase function
Source: Nat Commun. 2022 May 3;13:2416. doi: 10.1038/s41467-022-29928-8 (PMC9065133; doi:10.1038/s41467-022-29928-8)
Supplement: Supplementary file 5 — Reporting Summary [file 41467_2022_29928_MOESM5_ESM.pdf]

nature portfolio

Corresponding author(s): Cherepanov, Engelman, Yardimci, Lyumkis

Last updated by author(s): Jan 17, 2012

Reporting Summary

Nature Portfolio wishes to improve the reproducibility of the work that we publish. This form provides structure for consistency and transparency in reporting. For further information on Nature Portfolio policies, see our Editorial Policies and the Editorial Policy Checklist.

Statistics

For all statistical analyses, confirm that the following items are present in the figure legend, table legend, main text, or Methods section.

n/a	Confirmed	
☐	☒	The exact sample size (*n*) for each experimental group/condition, given as a discrete number and unit of measurement
☐	☒	A statement on whether measurements were taken from distinct samples or whether the same sample was measured repeatedly
☐	☒	The statistical test(s) used AND whether they are one- or two-sided<br>*Only common tests should be described solely by name; describe more complex techniques in the Methods section.*
☒	☐	A description of all covariates tested
☒	☐	A description of any assumptions or corrections, such as tests of normality and adjustment for multiple comparisons
☐	☒	A full description of the statistical parameters including central tendency (e.g. means) or other basic estimates (e.g. regression coefficient) AND variation (e.g. standard deviation) or associated estimates of uncertainty (e.g. confidence intervals)
☐	☒	For null hypothesis testing, the test statistic (e.g. *F*, *t*, *r*) with confidence intervals, effect sizes, degrees of freedom and *P* value noted<br>*Give P values as exact values whenever suitable.*
☒	☐	For Bayesian analysis, information on the choice of priors and Markov chain Monte Carlo settings
☒	☐	For hierarchical and complex designs, identification of the appropriate level for tests and full reporting of outcomes
☒	☐	Estimates of effect sizes (e.g. Cohen's *d*, Pearson's *r*), indicating how they were calculated

Our web collection on statistics for biologists contains articles on many of the points above.

Software and code

Policy information about availability of computer code

Data collection	Cryo-EM image collection software as described in Methods section: EPU, version 1.9 (Thermo Fisher) and Leginon version 3.0.<br>TIRF data were acquired using NIS-Elements software version 4 (Nikon).<br>Real-time PCR data were acquired using QuantStudio 7 Flex system and software version 1.3 (Applied Biosystems).<br>Luciferase measurements were done using EnVision version 2102 software version 1.13 (Perkin Elmer).<br>Flow cytometry were acquired using BD Diva software version 6.0 (BD Biosciences).<br>Integration site sequencing was done using Illumina HiSeq-4000 platform and software, operated by GenWiz.<br>Chromatograms were recorded using Unicorn version 5.31 software (GE Healthcare).
Data analysis	Data analysis and validation was conducted using commercially or publicly available software (all references are given in the manuscript).<br>Motion correction MotionCor2 version 1.4.0; CTF estimation: CFFIND4 version 4.1.5<br>Cryo-EM image processing and 3D volume reconstruction: Relion version 2.1 and version 3.1 (as described) and cryoSPARC version 3.2.0, and Eman2 version 2.07.<br>Cryo-EM map post-processing: Phenix version dev-4213-000 and DeepEMhancer (using original published version: https://github.com/rsanchezgarc/deepEMhancer).<br>Cryo-EM structure real-space refinement: Coot version 0.9.8 and Phenix version dev-4213-000 .<br>Final model validation: MolProbity version 4.5 and Phenix version dev-4213-000 .<br>MALLS data were analyzed using ASTRA version 6.1 software (Wyatt Technology).<br>Real-time PCR data were analyzed using QuantStudio 7 software.<br>Flowcytometry data were analyzed using FlowJo version 13.<br>Photobleaching data were analyzed in Matlab version R2019a and Prism version 7 (GraphPad).<br>Integration sites were mapped and analyzed using BWA version 0.7.12-r1039, SAMtools version 1.2 and BEDtools version 2.25.0.<br>Statistical tests were performed in R version 4.0.4, MicroSoft Excel version 16.56, Prism version 7, and data were plotted with Prism version 7.

Sequence logos were generated using WebLogo version 3.

For manuscripts utilizing custom algorithms or software that are central to the research but not yet described in published literature, software must be made available to editors and reviewers. We strongly encourage code deposition in a community repository (e.g. GitHub). See the Nature Portfolio guidelines for submitting code & software for further information.

Data

Policy information about availability of data

All manuscripts must include a data availability statement. This statement should provide the following information, where applicable:

- Accession codes, unique identifiers, or web links for publicly available datasets
- A description of any restrictions on data availability
- For clinical datasets or third party data, please ensure that the statement adheres to our policy

Final cryo-EM reconstructions were deposited with the EMDB under accession codes EMD-26322 (TCC) and EMD-14453 (STC) and fitted coordinates with the PDB under accession codes 7U32 (TCC) and 7Z1Z (STC).

Illumina sequencing data along with locations of mapped MVV integration sites were deposed with the Gene Expression Omnibus database under accession code GSE196042.

The following genome assemblies were used: human hg38 (https://hgdownload.soe.ucsc.edu/goldenPath/hg38/) and sheep oviAri4 (https://hgdownload.soe.ucsc.edu/goldenPath/oviAri4).

HEK293T gene expression data were from Gene Expression Omnibus database entry GSE11892 (https://www.ncbi.nlm.nih.gov/geo/query/acc.cgi?acc=GSE11892). Sheep gene expression data were from reference 109 (link to data: https://doi.org/10.1371/journal.pgen.1006997.s009)

The following protein data bank entries were used: 3HPH (https://www.rcsb.org/structure/3HPH), 5M0R (https://www.rcsb.org/structure/3HPH), 3OY9 (https://www.rcsb.org/structure/3OY9), 4BAK (https://www.rcsb.org/structure/4BAK), 6VOY (https://www.rcsb.org/structure/6VOY), 5EJK (https://www.rcsb.org/structure/5EJK)

Field-specific reporting

Please select the one below that is the best fit for your research. If you are not sure, read the appropriate sections before making your selection.

☒ Life sciences ☐ Behavioural & social sciences ☐ Ecological, evolutionary & environmental sciences

For a reference copy of the document with all sections, see nature.com/documents/nr-reporting-summary-flat.pdf

Life sciences study design

All studies must disclose on these points even when the disclosure is negative.

Sample size

No statistical methods were used to predetermine sample size. Sample size for the cryo-EM studies was determined by the availability of microscope time and to ensure unambiguous modeling of the intasome structures. Sample sizes of the cryo-EM data sets are given in Supplementary Table 1. Given the severe phenotypes of the mutants studied here, strong statistical power was not required, as long as results of functional assays are reproducible. Experiments were either repeated independently at least three times as described in the manuscript. Overall, the design described here is sufficient to detect meaningful biological differences with significant reproducibility.

Data exclusions

IN mutant aggregation precluded MALLS analysis in some conditions (for example, H12N MVV IN at 8 mg/ml); these data were not reported in Fig. 3C.

Replication

Experiments were repeated, as detailed in the figure legends. The data described in the manuscript are reproducible.

Randomization

Randomization is built into cryo-EM image processing in Relion and CryoSPARC, which use independent random subsets of particle images. However, randomization is not applicable to model building, nor was it used in functional studies, since specific integrase or virus mutants were compared. Randomization is a foundation of clinical trials when assessment of individuals who will receive test drug versus placebo is randomly assessed, which is critical to ensure that study outcome is independent from biased study design. In rather sharp contrast, ours was a hypothesis-based study that used the outcomes of engineered protein and virus behaviors to either support or refute the hypothesis, which then informed the design of additional mutant test constructs. In all cases, there were prescribed mutants to test. Random allocation of different test proteins or viruses to separate subsets for tests did not apply and would not have made sense.

Blinding

Cryo-EM data collection is inherently blinded, as the molecular structure (cryo-EM map) is interpretable to a human only after averaging many individual particle images. Experiments were generally not blinded. Blinding is appropriate when subjectivity or bias, conscious or not, can be introduced by the human observer in a comparison (e.g., placebo vs active drug, visual scoring of microscopic images such as staining intensity or disease pathology, etc). In the present work, measurements were with done with quantitative assays, using instruments that generate numerical values with statistical bounds and were not subject to observer bias or subjectivity as they were made. Structures themselves are not assessable blindly, nor is this ever done as far as we are aware, since knowledge of the molecule and its virological and biochemical roles are essential to an informed interpretation.

Reporting for specific materials, systems and methods

We require information from authors about some types of materials, experimental systems and methods used in many studies. Here, indicate whether each material, system or method listed is relevant to your study. If you are not sure if a list item applies to your research, read the appropriate section before selecting a response.

Materials & experimental systems

n/a	Involved in the study
☐	☒ Antibodies
☐	☒ Eukaryotic cell lines
☒	☐ Palaeontology and archaeology
☒	☐ Animals and other organisms
☒	☐ Human research participants
☒	☐ Clinical data
☒	☐ Dual use research of concern

Methods

n/a	Involved in the study
☒	☐ ChIP-seq
☒	☐ Flow cytometry
☒	☐ MRI-based neuroimaging

Antibodies

Antibodies used

The following primary antibodies were used: rabbit polyclonal anti-LEDGF/p75 (Bethyl Laboratories; product code A300-848A; Lot A300-848-1), rabbit polyclonal anti-HRP2 (Novus Bio; product code NBP2-47438; Lot R40643), affinity-purified polyclonal rabbit anti-MVV capsid/p24 antibody (Thermo Fisher Scientific; custom product code HAB2110A; Lot AB2670), and horseradish peroxidase-conjugated rabbit monoclonal anti-beta-actin antibody cone 13E5 (Cell Signaling Technology, product code 5125; Lot 6). Blots probed with anti-LEDGF/p75 (diluted 1:5,000 in phosphate buffered saline, supplemented with 0.05% Tween-20 (PBST)), anti-HRP2 (diluted 1:1,000 in PBST), and anti-capsid/p24 antibodies (1:5,000).

Secondary antibodies were horseradish peroxidase-conjugated goat anti-rabbit IgG antibody (BioRad; product code 1706515; Lot 64126042; diluted 1:10,000 in PBST) or IRDye 800CW conjugated goat anti-rabbit IgG antibody (LI-COR; product code 925-32211; Lot C90220-06; diluted 1:2,000 in PBST).

Validation

Commercially available antibodies:
https://fortis-datasheets.s3.us-east-2.amazonaws.com/A300-848A-1.pdf
https://www.novusbio.com/products/hrp-2-antibody_nbp2-47438
https://www.cellsignal.co.uk/products/antibody-conjugates/b-actin-13e5-rabbit-mab-hrp-conjugate/5125
https://www.agilent.com/cs/library/packageinsert/public/SSP0399CEEFG_01.pdf
https://www.licor.com/documents/rfm2hw40wf33p06f3ndjrcorwi5usbft

MVV CA/p24 antibody was validated in this work (Supplementary Fig. 12).

Eukaryotic cell lines

Policy information about cell lines

Cell line source(s)

The human cell lines LKO and parental HEK293T have been published (https://journals.asm.org/doi/10.1128/JVI.01397-14). Derivation of LHKO and LHKO-LEDGF is described in the manuscript.

The clonal cell line CPT3 (obtained from Massimo Palmarini, University of Glasgow) was derived through limited dilution of CPT-Tert, ovine choroid plexus cells immortalized by co-expression of simian virus 40 large T antigen and human telomerase (https://journals.asm.org/doi/10.1128/JVI.00029-10). The derivatives CPT3-LKO and CPT3-LHKO were generated in the current work, as described in Methods section.

Authentication

CPT-Tert cells were authenticated by sequencing of ovine cDNAs (https://journals.asm.org/doi/10.1128/JVI.00029-10). Knockout cell lines were authenticated by Western blotting (Supplementary Figs 8a and 9a). No other cell line authentication was performed in this study.

Mycoplasma contamination

The cells were confirmed to be free of mycoplasma contamination by the Francis Crick Institute Tissue Culture Scientific Technology Platform using commercial kits.

Commonly misidentified lines (See ICLAC register)

No commonly misidentified cell lines were used in this study.
